# Approaches to Study Gap Junctional Coupling

**DOI:** 10.3389/fncel.2021.640406

**Published:** 2021-03-10

**Authors:** Jonathan Stephan, Sara Eitelmann, Min Zhou

**Affiliations:** ^1^Institute of Neurobiology, Heinrich Heine University Düsseldorf, Düsseldorf, Germany; ^2^Department of Neuroscience, Wexner Medical Center, Ohio State University, Columbus, OH, United States

**Keywords:** **patch** clamp, paired recordings, astrocyte syncytial isopotentiality, tracer coupling, wide field imaging

## Abstract

Astrocytes and oligodendrocytes are main players in the brain to ensure ion and neurotransmitter homeostasis, metabolic supply, and fast action potential propagation in axons. These functions are fostered by the formation of large syncytia in which mainly astrocytes and oligodendrocytes are directly coupled. Panglial networks constitute on connexin-based gap junctions in the membranes of neighboring cells that allow the passage of ions, metabolites, and currents. However, these networks are not uniform but exhibit a brain region-dependent heterogeneous connectivity influencing electrical communication and intercellular ion spread. Here, we describe different approaches to analyze gap junctional communication in acute tissue slices that can be implemented easily in most electrophysiology and imaging laboratories. These approaches include paired recordings, determination of syncytial isopotentiality, tracer coupling followed by analysis of network topography, and wide field imaging of ion sensitive dyes. These approaches are capable to reveal cellular heterogeneity causing electrical isolation of functional circuits, reduced ion-transfer between different cell types, and anisotropy of tracer coupling. With a selective or combinatory use of these methods, the results will shed light on cellular properties of glial cells and their contribution to neuronal function.

## Introduction

**FIGURE 1:**
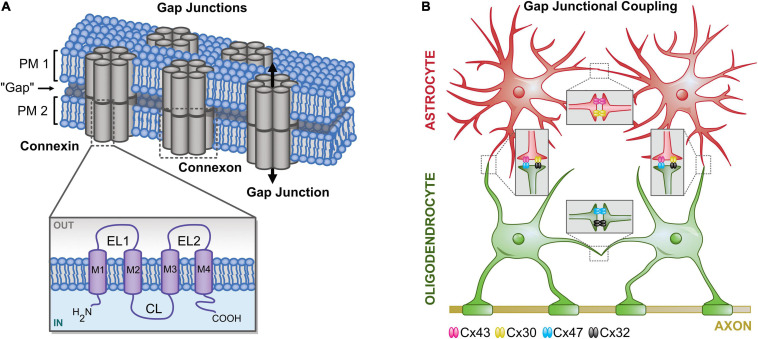
Principle of gap junctional coupling. **(A)** Structural organization of gap junctions. Gap junctions are integral membrane proteins that connect the cytosol of neighboring cells. Two pairs of connexons (hexamers of connexins; Cx) form a pore enabling diffusion for ions and small molecules. EL, extracellular loop; CL, cytoplasmic loop; M, transmembrane helix. **(B)** (Pan-/)glial coupling. Astrocytes mainly express Cx43 and Cx30, whereas oligodendrocytes mainly express Cx47 and Cx32. Homotypic gap junctions couple astrocytes (Cx43:Cx43, Cx30:Cx30) and oligodendrocytes (Cx47:Cx47, C30:Cx30). Heterotypic gap junctions are formed by different connexons connecting astrocytes and oligodendrocytes (Cx43:Cx47, Cx30:Cx32).

Gap junction channels connect the cytosol of neighboring cells and allow the exchange of ions and small molecules, such as metabolites (Giaume et al., 2010, 2020). Gap junctions form, when two connexons in the membrane of neighboring cells align. Connexons in turn are hexamers that are formed by connexins (Cx; Figure 1A). There are 21 Cxs identified so far of which 11 are expressed by neurons and glial cells in the CNS that differ in their mass (Bedner et al., 2012) and expression profile (including developmental and cell type specificity) (Dahl et al., 1996; Kunzelmann et al., 1999; Nagy et al., 1999; Nagy and Rash, 2000; Augustin et al., 2016; Wadle et al., 2018). The composition of gap junctions with different connexons and Cxs defines their properties, for example selectivity and permeability (Bukauskas and Verselis, 2004; Harris, 2007; Abbaci et al., 2008). In addition, post-translational modifications further regulate gap junctional communication (Goodenough and Paul, 2009; Axelsen et al., 2013; Aasen et al., 2018). Homocellular coupling between astrocytes and oligodendrocytes is mediated by homotypic gap junctions (Figure 1B; Giaume and Theis, 2010; Maglione et al., 2010). Furthermore, heterocellular (panglial) coupling is observed between both cell types using heterotypic gap junctions (Wasseff and Scherer, 2011; Griemsmann et al., 2015; Augustin et al., 2016; Moshrefi-Ravasdjani et al., 2017; Claus et al., 2018; Wadle et al., 2018; Xin et al., 2019). In the corpus callosum, astrocytes and NG2 glia form panglial networks (Moshrefi-Ravasdjani et al., 2017). However, in many other brain regions—like the hippocampus, the cortex, and the medial nucleus of the trapezoid body—NG2 glia are neither tracer nor electrically coupled to other glial cells (Wallraff et al., 2004; Houades et al., 2008; Muller et al., 2009; Xu et al., 2014; Bedner et al., 2015; Griemsmann et al., 2015).

Gap junctional coupling is important for homeostasis of K^+^ (Wallraff et al., 2006; Pannasch et al., 2011; Breithausen et al., 2020; MacAulay, 2020), Na^+^ (Langer et al., 2012; Augustin et al., 2016; Moshrefi-Ravasdjani et al., 2017; Wadle et al., 2018), Cl^–^ (Egawa et al., 2013), and neurotransmitters (Pannasch et al., 2011; Chaturvedi et al., 2014). Furthermore, regulated gap junctional coupling is mandatory for activity-dependent redistribution of metabolites, such as glucose (Cruz et al., 2007; Rouach et al., 2008; Giaume et al., 2010). In addition, Cx expression and gap junctional communication are altered under pathophysiological conditions (Cotrina et al., 1998; Nakase et al., 2006; Giaume et al., 2010, 2019, 2020; Xu et al., 2010; Bedner et al., 2015; Lee et al., 2016; Wang Q. et al., 2020). Thus, the basic knowledge about glial gap junctional communication is fundamentally important for our further understanding of the complex neuron-glia interaction in healthy and diseased brains.

Astrocytes can be identified by several approaches. Many astrocytes express marker proteins such as GFAP, S100β, GLAST, or GLT-1 (Eng et al., 2000; Zhou et al., 2006; Kafitz et al., 2008; Schreiner et al., 2014). However, there are subpopulations of astrocytes, which for example do not express GFAP (Kafitz et al., 2008). Additionally, a fraction of NG2 glia are characterized by expression of GFAP and S100β (Nolte et al., 2001; Matthias et al., 2003; Karram et al., 2008). In 2004, the red fluorescent dye sulforhodamine (SR) 101 was introduced as a marker for astrocytes (Nimmerjahn et al., 2004), which allows the *a priori* identification of classical astrocytes in various brain regions, for example, in hippocampus, cortex, and auditory brainstem (Nimmerjahn et al., 2004; Kafitz et al., 2008; Stephan and Friauf, 2014; Ghirardini et al., 2018). Additionally, the use of SR101 is particularly advantageous in astrocyte imaging experiments as it can be combined with ion sensitive dyes such as Fura-2 and sodium-binding benzofuran isophthalate (SBFI; Kafitz et al., 2008; Langer et al., 2012).

Another approach for *a priori* identification of astrocytes is the utilization of reporter mice, such as GFAP-eGFP mice (Nolte et al., 2001). However, the transcript labels only a subset of astrocytes (Nimmerjahn et al., 2004) and, moreover, the transcript is also weakly expressed by NG2 glia (Matthias et al., 2003). Alternatively, ALDH1L1-eGFP mice can be used to identify astrocytes (Heintz, 2004; Yang et al., 2011). These reporter mice exhibit a more accurate labeling pattern of astrocyte populations (Cahoy et al., 2008). Aside this, reporter mice are available to *a priori* identify other glia, such as oligodendrocytes (PLP-GFP mice; Fuss et al., 2000) or NG2 glia (NG2-eYFP mice; Karram et al., 2008). It is worth mentioning that all these reporter mice are suitable to be combined with imaging of ion-sensitive dyes (Moshrefi-Ravasdjani et al., 2017).

A hallmark of classical astrocytes is their large K^+^ conductance (Stephan et al., 2012), which results in a highly negative membrane potential (Zhou et al., 2006; Kafitz et al., 2008). Further, astrocyte properties are less constant as the expression of many astrocyte-typical proteins is regulated during early postnatal development (Felix et al., 2020b). For example, the expression of inwardly rectifying k^+^ channels (Kir_4.1_) and two-pore domain K^+^ channels (K_2P_) increases (Seifert et al., 2009; Nwaobi et al., 2014; Lunde et al., 2015; Moroni et al., 2015; Olsen et al., 2015) causing a strong decrease in membrane resistance (*R*_*M*_; Zhou et al., 2006; Kafitz et al., 2008; Stephan et al., 2012; Zhong et al., 2016). Simultaneously, the detectable amount of K_*V*_ channel-mediated currents decreases, which together alters the astrocytic current-voltage relationship from non-linear to linear (Zhou et al., 2006; Kafitz et al., 2008; Zhong et al., 2016). In contrast to NG2 glia, astrocytes do not express Na_*V*_ channels (Matthias et al., 2003; Zhou et al., 2006; Kafitz et al., 2008; Zhong et al., 2016).

## Analysis of Gap Junctional Coupling

There are many different techniques available to study gap junctions. These include but are not limited to whole-cell patch-clamp (paired recordings, analysis of isopotentiality, tracer injection), genetic approaches (FRAP, PARIS, StarTrack, transgenic mice), imaging of ion-sensitive dyes (e.g., SBFI), and expression studies (immunohistochemistry, western blotting) (Abbaci et al., 2008; Giaume and Theis, 2010; Bedner et al., 2012; Langer et al., 2012; Griemsmann et al., 2015; Droguerre et al., 2019; Eitelmann et al., 2019; Gutierrez et al., 2019; Wu et al., 2019; Du et al., 2020; McCutcheon et al., 2020). Here, we focus on approaches to analyze gap junctional communication that can be implemented easily in most electrophysiology and imaging laboratories.

### Patch Clamp-Based Approaches

Electrophysiological methods are commonly used to detect gap junctional coupling of cells. In 1966, the first evidence that astrocytes are intercellularly coupled was provided by an electrophysiological study of amphibian optic nerve by Kuffler et al. (1966). In their report, an elegant triple-sharp-electrode recording mode was used to reveal a “low-resistance connection” between neuroglia, which we know now as the gap junctional coupling of fibrous astrocytes in optic nerves. This electrophysiological method was continually used until the 1980s for glial physiology study. For example, Kettenmann and Ransom used it to record cultured astrocytes and oligodendrocytes, confirming that gap junctions were indeed the molecular identities for the functional coupling of these glial subtypes (Kettenmann and Ransom, 1988; Ransom and Kettenmann, 1990).

The advent of patch-clamp in the 1990s ushered electrophysiological studies into a new era. Since the patch-clamp system is able for simultaneous current injection and membrane potential recording, now only two electrodes are used for paired recording. Here, we will limit our discussion to this advanced paired recording mode and its application to analyze the functional connectivity of neighboring astrocytes. Until now, this technique has been used by several research laboratories for study of gap junctional coupling in native astrocytes in brain slices and freshly isolated astrocytes (Muller et al., 1996; Meme et al., 2009; Xu et al., 2010, 2014; Ma et al., 2016; Zhong et al., 2016; Kiyoshi et al., 2018). These studies demonstrated the paired recording mode as a highly sensitive method for revealing the functional coupling of astrocytes *in situ* and in pairs of freshly dissociated astrocytes. To address the electrical role of gap junctional coupling for astrocyte syncytium, a single electrode method was developed in 2016 with details described in the following “Astrocyte Syncytial Isopotentiality” section. Together with computational modeling, this method allows for monitoring dynamical changes in the coupling strength of an astrocyte syncytium (Ma et al., 2016; Kiyoshi et al., 2018). Another way to assess gap junctional coupling is to add a tracer to the internal solution of patch pipettes and to visualize gap junction-coupled cells. In the following section, we will first discuss the rationale, application, advantage, and limitation of the commonly used paired recording model and the newly developed syncytial isopotentiality measurement. Thereafter, we will highlight, how the addition of gap junction-permeable tracers to the pipette solution can be used to visualize cell-to-cell coupling and to subsequently analyze the topography of tracer-coupled networks.

#### Paired Recording Mode

**FIGURE 2:**
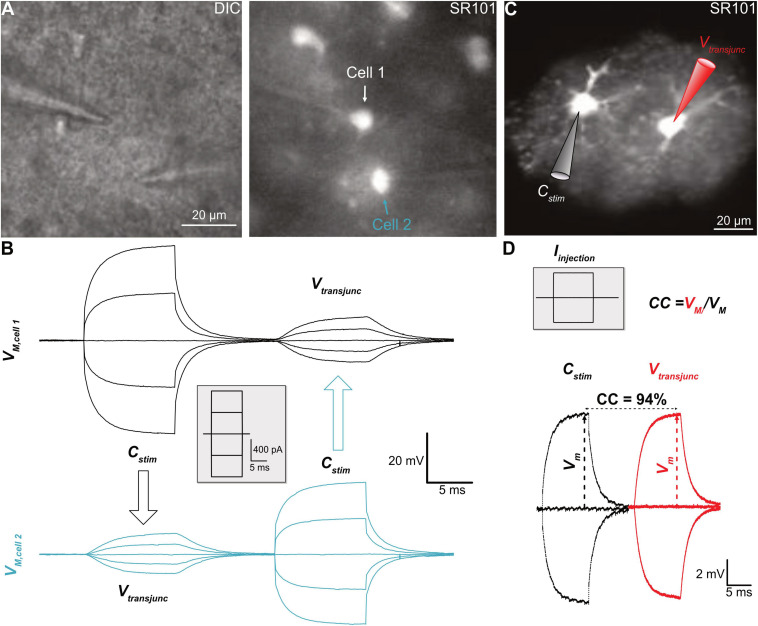
Analysis of electrical coupling. **(A)** Paired recording of two neighboring astrocytes from hippocampal CA1 region. **(B)** In whole-cell current-clamp mode, the current steps, shown in the inset, were alternately delivered to one of the cells in a pair, termed the stimulated cell (*C*_stim_), and that induced membrane potential changes in the *C*_stim_ meanwhile induced transjunctional voltages (*V*_*transjunc*_) in the coupled neighbor. **(C)** A pair of freshly dissociated hippocampal astrocytes, identified by their SR101 staining, was recorded with K^+^ free/Na^+^ containing electrode solution. **(D)** The current injections into one of them induced comparable size of the membrane voltage displacements in both cells, indicating a strong coupling coefficient (*CC*) at an estimated value of 94%. Panels **(A)** and **(B)** modified from Zhong et al. (2016), and Panels **(C)** and **(D)** modified from Ma et al. (2016).

The rationale behind the design of the paired recording model lies in the basic properties of gap junction channels. Gap junctions are large aqueous pores, 8–16 Å, filled with electrolytes that make them ideal electrical conductors for the flow of ionic currents between coupled cells (Weber et al., 2004). In the adjacent cells, the easiness of the ionic current flow is determined by the number of gap junctions aggregated on the plaques in the interface of the adjacent cells, or the intercellular gap junctional resistance (*R*_gap_). Accordingly, the paired recording mode is designed to examine the *R*_gap_ through the passing of injected currents in one of the two coupled cells and to measure subsequently the size of remaining transjunctional voltage arriving at the second cell (Figures 2A,B; Bennett, 1966; Bennett et al., 1991; Ma et al., 2016). There are unique characteristics associated with paired recordings. It is known that the gap junctions formed by different Cxs differ in their permeability properties for endogenous compounds, therefore, differentially regulate the intercellular transfer of metabolites, i.e., glucose, and signaling molecules, such as ATP, glutamate, and IP_3_ (Goldberg et al., 1999, 2002; Niessen et al., 2000). It is also known that Cxs vary in pore size and conductivity (∼30 to ∼300 pS) (Hille, 2001). However, the Cxs ubiquitously exhibit high selectivity to the major intracellular monovalent cation K^+^ and Na^+^ in the order of K^+^ > Na^+^ (Wang and Veenstra, 1997), and these ions are charge carriers that mediate current flow between coupled cells. Thus, independent of Cx isoforms, transjunctional voltage measurements stand out as an universal readout of gap junctional coupling (Veenstra et al., 1995; Veenstra, 1996). Additionally, paired recordings are featured by their high detection sensitivity conferred by the state-of-the-art electronic engineering technology of the patch-clamp amplifier, allowing detection of the ionic currents at the picoampere scale.

Experimentally, two adjacent astrocytes are sequentially recorded in whole-cell mode (Figures 2A,B). Subsequently, the current can be alternately injected into one of the recorded cells, defined as the stimulated cell (*C*_stim_.), whereas the transjunctional voltage is recorded in the neighboring recipient cell (*C*_reci_.). Although the *R*_gap_ can be calculated from the basic membrane properties and the current-induced membrane voltages, the coupling strength is commonly expressed as the coupling coefficient (*CC*)—the ratio of voltages measured from the *C*_reci_./*C*_stim_. The higher a *CC* value, the stronger the cell-to-cell coupling (Bennett, 1966; Bennett et al., 1991; Ma et al., 2016).

A major advantage of paired recordings is their high sensitivity, which is determined by the open probability that is universally high among gap junctions in the range of 0.6–0.9; whereas the sensitivity of tracer coupling is mostly determined by the pore selectivity that varies among gap junctions (Nielsen et al., 2012). Consequently, in simultaneous transjunctional voltage and dye coupling measurements, it is common to see measurable transjunctional voltages in an absence of detectable dye coupling (Murphy et al., 1983; Ransom and Kettenmann, 1990; Sontheimer et al., 1991; Xu et al., 2010). An example that paired recordings have a higher sensitivity was shown in a study carried out in hippocampal astrocytes *in situ* (Xu et al., 2010). Incubation of brain slices with a gap junction inhibitor, meclofenamic acid (MFA, 100 μM), was able to inhibit astrocyte gap junctional coupling by 99% (Ma et al., 2016). Interestingly, in the presence of 100 μM MFA, the cross-diffusion of two tracers, Lucifer Yellow (LY) and biocytin, separately loaded into two recording electrodes, were completely inhibited, whereas the transjunctional voltages remained intact (Xu et al., 2010). In the barrel cortex, the Cx43 expression was found to be enriched within the barrels, but largely absent in the inter-barrel space (septa), and this was associated with a restricted dye coupling inside the barrels (Houades et al., 2006). To examine whether such segregated dye coupling indeed indicates a complete absence of gap junctional of astrocytes between barrels and their surrounding septa, the paired recording was carried out between a pair of astrocytes, one inside another outside of the barrel. The results showed that in the transjunctional voltage measurement, astrocytes do communicate inside and outside of the barrels and, therefore, are gap junctional coupled into a syncytium (Kiyoshi et al., 2018). Therefore, the existence of gap junctional coupling can be more sensitively inspected by this method.

Nevertheless, there are also technical challenges and limitations. First of all, still, only a handful of experimenters successfully employed paired recordings in their research. The technical complexity of paired recordings to monitor junctional coupling has limited more researchers to take advantage of this powerful tool in their glial physiology study. The second obstacle that impedes the application of paired recordings is the low *R*_*M*_ of astrocytes at an estimated level of 6.4 MΩ (Du et al., 2015). As a result, the “leaky” membrane shunts most of the injected currents, making it impossible to estimate the current passing through gap junctions resulting in erroneous *CC* calculation (Du et al., 2020). To make the matters worse, astrocytes are coupled into a syncytial network; each astrocyte is typically directly coupled to 7–9 nearest neighbors across the brain (Xu et al., 2010; Ma et al., 2016; Kiyoshi et al., 2018). Consequently, the injected currents to one of the recorded astrocytes should be redistributed into coupled astrocytes at unknown ratios (in a parallel electrical circuit) (Cotrina et al., 1998). Therefore, the leaking membrane and syncytial coupling make it next to impossible to calculate the actual *CC* in brain slice recordings. Consequently, rather low *CC* values in the range from 1.6% to 5.1% were reported from hippocampal astrocytes *in situ* (Meme et al., 2009; Xu et al., 2010).

To solve this problem, the innovative use of paired recordings was applied to freshly dissociated pairs of astrocytes to avoid extensive coupling. To circumvent the shunt of injected currents through abundantly expressed membrane K^+^ channels, the physiological K^+^ content in the electrode solution was substituted equimolarly by Na^+^; hence, the electrode Na^+^ equilibrating with the recorded pair of astrocytes will not leak through the membrane K^+^ channels, and measured currents will therefore better reflect junctional coupling (Figures 2C,D). Under these conditions, a strong coupling *CC* of 94% was revealed. Based on this *CC*, there was an estimate of 2.000 gap junctions aggregated at the interface of two neighboring astrocytes, and a calculated *R*_gap_ at 4.3 MΩ (Ma et al., 2016), which is even lower than the astrocytic *R*_*M*_ of 6.4 MΩ (Du et al., 2015). These results together indicate that the electrical barrier between astrocytes is nearly absent. Recently, ultrastructural details of astrocyte-astrocyte contacts have been revealed that explain how such a low inter-astrocytic resistance could be biophysically achieved (Kiyoshi et al., 2020).

In summary, the rationale for paired recordings is based on an uniform feature of high open probability of gap junction channels for two intracellular monovalent cations, K^+^ and Na^+^. Therefore, it offers a rather universal readout to study the functional gap junctional coupling at high sensitivity. For brain slice studies, however, the paired recordings are most valuable for inspecting the existence of functional gap junctional coupling, but are of limited value for quantitative analysis of the *CC* between astrocytes due to the low *R*_*M*_ and syncytial coupling. Combinatory use of freshly dissociated pairs of astrocytes and non-physiological Na^+^ or Cs^+^ electrode solution is a powerful alternative to circumvent the above obstacles.

#### Astrocyte Syncytial Isopotentiality

As mentioned above, the *R*_gap_ between astrocytes is even lower than astrocytes’ *R*_*M*_, suggesting that two neighboring astrocytes are able to constantly equalize their membrane potentials and therefore electrically behave as one cell. By extension, the gap junction coupled astrocytes should then be able to balance their membrane potentials to comparable levels so that a syncytial isopotentiality could be achieved. In fact, this possibility was postulated in the past (Muller, 1996) and was discussed by Richard Orkand and his colleagues to be a necessity for the operation of K^+^ spatial buffering in the brain (personal communication with Dr. Serguei Skatchkov). This syncytial isopotentiality was experimentally demonstrated in 2016 (Ma et al., 2016), and a system-wide existence of this feature in the astrocyte networks was confirmed soon after that (Huang et al., 2018; Kiyoshi et al., 2018; Wang W. et al., 2020).

**FIGURE 3:**
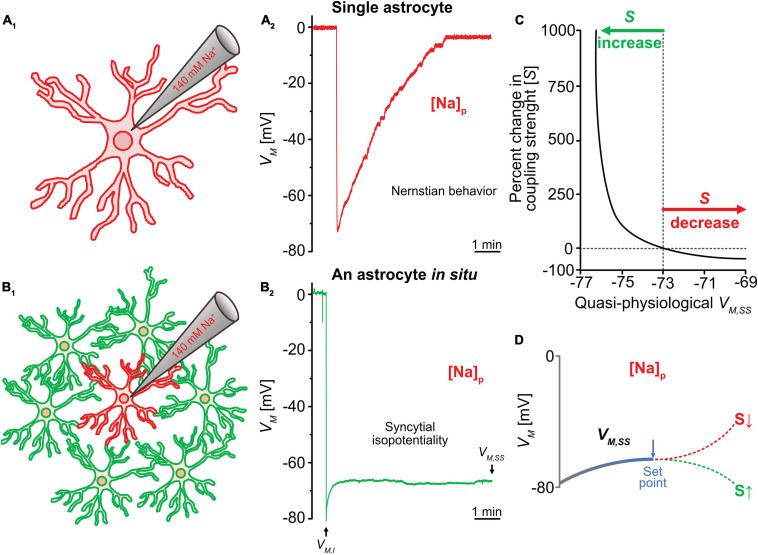
Analysis of syncytial isopotentiality. **(A)** In a single freshly dissociated astrocyte, the K^+^ free/Na^+^ containing electrode solution ([Na^+^]_*p*_) progressively substituted the endogenous K^+^ content in whole-cell recording, leading to a *V*_*M*_ depolarization toward 0 mV as predicated by Nernstian equation. **(B)** The *V*_*M*_ recorded from an astrocyte *in situ* with the [Na^+^]_*p*_ disobeys the Nernstian prediction. Instead, the steady-state *V*_*M*_ (*V*_*M*,__*SS*_) maintained at a quasi-physiological level as a result of voltage compensation by the physiological *V*_*M*_ of the coupled neighbors. **(C)** The relationship between *V*_*M*,__*SS*_ and coupling strength (*S*) predicted by computational modeling. **(D)** According to this modeling shown in **(C)**, the *V*_*M*,__*SS*_ can be shifted toward a more hyperpolarizing *V*_*M*_ of neighboring astrocytes due to a stronger syncytial coupling (*S*) or due to a weaker coupling strength (S), the *V*_*M*,__*SS*_ toward the GHK predication for the *V*_*M*_ of [Na^+^]_*p*_ at 0 mV. *V*_*M,I*_ stands for initial *V*_*M*_ recorded immediately after break-in of cell membrane, reflecting the resting *V*_*M*_ of the recorded astrocyte. Figure modified from Kiyoshi et al. (2018).

The rationale for the methodological design is based on a basic feature of astrocytes. Physiologically, an astrocyte behaves as a perfect K^+^ electrode (Kuffler et al., 1966; Ransom and Goldring, 1973). Therefore, one can experimentally substitute the intracellular K^+^ concentration ([K^+^]_*i*_) through dialysis of the recorded cell with electrode solution containing equimolar (i.e. 140 mM) Na^+^ (or Cs^+^) (Ma et al., 2016; Wang W. et al., 2020). This, in turn, alters the *V*_*M*_ of the recorded astrocyte from K^+^ equilibrium potential (*E*_*K*_, -80 mV) to Na^+^ equilibrium potential (*E*_*Na*_, ∼ 0 mV) according to the prediction from the Goldman-Hodgkin-Katz (GHK) equation. In single freshly dissociated astrocytes, the *V*_*M*_ indeed shifts to ∼ 0 mV (Figure 3A) (Kiyoshi et al., 2018). However, should the *R*_gap_ be sufficiently low, the associated syncytium can then instantaneously act to compensate for the loss of physiological membrane potential (*V*_*M*_) in the recorded cell, and the level of the compensation should be determined by the coupling strength and the number of directly nearest coupled neighbors (Ma et al., 2016; Kiyoshi et al., 2018).

Experimentally, the syncytial isopotentiality can be detected by the substitution of endogenous K^+^ by a 140 mM Na^+^/K^+^-free electrode solution ([Na^+^]_*P*_) (Figures 3A,B) and recording the astrocyte in current-clamp mode. The breakthrough of the membrane patch shifted the *V*_*M*_ immediately to a resting membrane potential of -78 mV (Figures 3A,B). Over time, the *V*_*M*_ reaches a stationary level of -73 mV. This is in sharp contrast to the anticipated depolarization to 0 mV as predicted by the GHK equation for K^+^-free electrode solution (Figure 3A). The initial *V*_*M*_ recording (*V*_*M,I*_) reflects the true resting *V*_*M*_ of an astrocyte (Ma et al., 2016; Kiyoshi et al., 2018). In hippocampal astrocyte syncytium, the steady-state *V*_*M*_ (*V*_*M,SS*_) in [Na^+^]_*P*_ recordings is ∼5 mV more depolarized than *V*_*M,I*_.

##### The Ionic Mechanisms Engaged in the Establishment of *V_M, SS_*

To biophysically explain how the quasi-physiological *V*_*M*,*SS*_ is established, different size of syncytia, i.e., syncytia containing varying number of astrocytes, were used for *V*_*M, SS*_ recordings, and computational modeling was used to simulate the underlying ionic mechanisms (Ma et al., 2016). The rupture of an astrocyte with [Na^+^]_*p*_ initiates dialysis of Na^+^ to the recorded astrocyte and associated syncytium (Figure 3B_1_). In the recorded cell, the Na^+^ dialysis dissipates the endogenous K^+^ content hence abolishes the physiological *V*_*M*_ established by the across membrane K^+^ gradient. To the coupled syncytium, the Na^+^ dialysis generates a Na^+^ gradient and current flow across gap junctions that depolarizes neighboring astrocytes and hyperpolarizes the recorded cell. The latter is the major contributor to the quasi-physiological *V*_*M*,*SS*_. Additionally, the dissipation of K^+^ content in the recorded cell attracts an influx of K^+^ from neighboring astrocytes. The accumulated K^+^ has the potential to establish a hyperpolarizing potential in the recorded cell therefore additionally contributes to the totality of the quasi-physiological *V*_*M*,*SS*_ (Ma et al., 2016). As shown in Figure 3B_2_, these two ionic flows take approximately 5 min to reach a steady-state after rupture of whole-cell recording (Figure 3B_2_).

How does the strength of syncytial isopotentiality influence the intensity of the Na^+^ and K^+^ current flows? As noted above, in the [Na^+^]_*p*_ recorded astrocyte, there are two ionic current flows in opposite directions and collectively contribute to the measured V_M, SS_; the outward-going Na^+^ current from recorded cell to the coupled cells, and inward-going K^+^ current flow from the coupled astrocytes to the recorded cell. To simplify the analysis, we take the outward-going Na^+^ (*I*_*Na, out*_) to one of the coupled astrocytes as an example, the proximity of the potentials in these two cells can be expressed as the difference of the voltages between the Na^+^-loaded astrocyte (*V*_*Na*_) and a coupled neighbor (*V*_*N*_)

$$\left(V_{N}-V_{N\,a}\right)=R_{\text{gap}}*I_{\mathrm{Na},\mathrm{out}}$$

where (*V*_*N*_ - *V*_*Na*_) = 0 mV is a theoretically ideal isopotentiality achieved between the two cells. Assuming *I*_Na, out_ is a constant determined by its chemical gradient down the neighboring cell, then a higher strength of isopotentiality, i.e., lower (*V*_*N*_ – *V*_*Na*_), is correlated to a lower junctional resistance (*R*_gap_). Secondly, as (*V*_*N*_ – *V*_*Na*_) approaches the ideal isopotentiality of 0 mV, the *I*_Na, out_ also diminishes to ∼ 0 pA. By extension, closer proximity of potentials between the recorded cell and its associated astrocytes, the less outward-going Na^+^ current flow from [Na^+^]_*p*_ recorded astrocyte toward its associated syncytium.

Likewise, the intensity of the inward-going K^+^ current (*I*_*K, in*_) and syncytial coupling strength follows with the same relationship:

$$\left(V_{N}-V_{N\,a}\right)=R_{\text{gap}}*I_{K,i\,n}$$

where the inward-going K^+^ current is impeded by increasing proximity of the *V*_*Na*_ to *V*_*N.*_ It should be noted that K^+^ cannot be substantially buildup due to efflux of K^+^ through membrane K^+^ channels in the [Na^+^]_*p*_ recorded astrocyte; consequently, less hyperpolarizing potential can be built up to make a significant contribution to the recorded *V*_*M*,*SS*_.

In summary, we described a method that uses [Na^+^]_*p*_ to disrupt the continuity of a syncytial isopotentiality, and that in turn informs of the existence and the strength of the isopotentiality in an astrocyte syncytium. Biophysically, gap junctional ionic movement occurs during the equalization of the potential differences in a syncytium, therefore a strong syncytial isopotentiality means a less ionic movement inside a syncytium. Additionally, a larger syncytium has a greater capacity to minimize the ionic movement, which has been simulated in a computational model (Ma et al., 2016). Functionally, in the event of local extracellular environment changes, e.g., neuronal firing induced high K^+^, syncytial isopotentiality provides a sustained driving force to individual astrocytes for high efficient K^+^ uptake, spatial transfer and release of K^+^ to regions where neuronal activity is low (Terman and Zhou, 2019).

##### *V*_M, SS_ as a Functional Readout of Coupling Strength (S) of Syncytial Coupling

Based on the discussion above, the *V_M,SS_* is established and regulated by the *R*_gap_ and the number of astrocytes in a coupled syncytium, therefore can lean to a more hyperpolarizing *V*_*M*_ in the neighboring astrocytes, or a more depolarizing *V*_*M*_ established by intracellular Na^+^. Thus, the *V_M,SS_* serves as a dynamic readout of the strength of syncytial coupling. To be able to quantitatively correlate the changes of *V_M,SS_* with S, a computational model has been established (Figures 3C,D) where a stronger syncytial coupling leads to a stronger compensation of the *V_M,SS_* towards the physiological *V*_*M*_ of neighboring astrocytes established by 140 mV [K^+^]_*i*_, whereas a weaker coupling shifts the *V_M,SS_* toward GHK predicted *E*_*K*_ for the Na^+^ electrode solution. More details about the biophysical principles and assumptions used to build up this computational model are available in this publication (Kiyoshi et al., 2018). In addition, this model can be used for analysis of the dynamic change of coupling strength, for instance, during neuronal activation (Kiyoshi et al., 2018).

This method comprises several advantages. First, differing from paired recordings, *V_M,SS_* is measured from single electrode recordings. Second, this method allows for dynamic monitoring of the coupling strength of a syncytium over time. Third, this method can be incorporated with astrocyte syncytial anatomy studies. For example, ALDH1L1-eGFP reporter mice were use for *a priori* astrocyte identification and examination of syncytial isopotentiality across brain regions. Additionally, the recorded brain slices were then further processed with the tissue-clearing method (Susaki et al., 2014, 2015), i.e., depletion of the brain lipid content, for high-resolution confocal imaging study of the cellular morphology and spatial organization patterns of astrocytes (Kiyoshi et al., 2018). This study showed that in different layers of the visual cortex, the anatomy, in terms of cell density, interastrocytic distance and the number of the nearest neighbors vary in morphometric analysis. However, *S* does not differ between layers. Additionally, *S* of the visual cortex was found to be stronger than in the hippocampal CA1 region (Kiyoshi et al., 2018). Fourthly, this method can be incorporated with tracer coupling to map the directionality and spatial coupling of a syncytium (see also “Tracer Coupling”). For example, in the cerebellum, Bergmann glia and velate astrocytes are derived from the same progenitor pool but are strikingly different in their morphology (Kita et al., 2013). Bergmann glia are characterized by having their cell bodies arranged in rows alongside with the soma of Purkinje neurons and extension of their processes along the Purkinje cell layer toward the *pia* of the cerebellum. Velate astrocytes are cerebellar protoplasmic astrocytes that exhibit characteristic velate sheath processes and are more dispersed in arrangement (Chan-Palay and Palay, 1972). *S* is significantly weaker in Bergmann glia networks than those established by velate astrocytes at the granular layer. In the tracer coupling analysis, the injection of tracer revealed the coupling of Bergmann glia and velate astrocytes. Thus, despite a striking difference in syncytial anatomy, the syncytial isopotentiality occurs to syncytial networks established by both subtypes of astrocytes (Kiyoshi et al., 2018).

A limitation in this method is the inference of a syncytial isopotentiality based on biophysical principles and electrophysiological measurements. Significant progress has been made in the technique of genetically encoded voltage indicator (Kang et al., 2019). However, this state-of-the-art technique is still below the threshold to detect the subtle variation of voltages in an astrocyte syncytium, and therefore, future optimization of this technique is crucial to recruit advanced imaging techniques to study the physiology and pathology of astrocyte syncytial networks.

#### Tracer Coupling

Aside this direct measurement of cell-to-cell coupling, tracer coupling can be utilized to mimic diffusion within in the network. Tracers are usually loaded for several minutes into a single cell via the backfill of the patch pipette. Simultaneously, tracers diffuse within the gap junction network. The tracer concentration is highest in the patched cell and declines within the network with increasing distance as it diffuses. There are various tracers available comprising different advantages and disadvantages (Table 1; see alsoAbbaci et al., 2008). An often used tracer is LY (Kawata et al., 1983). It is a fluorescent dye that allows direct observation of diffusion within the network. Subsequently, LY labeling can be combined with immunohistochemistry to determine the identity of coupled cells (Binmoller and Muller, 1992;Konietzko and Muller, 1994). However, it comprises low permeability at gap junctions consisting of Cx30 (Rackauskas et al., 2007). As Cx30 expression increases during early postnatal development (Nagy et al., 1999;Griemsmann et al., 2015;Augustin et al., 2016;Wadle et al., 2018), LY will only highlight a fraction of coupling in more mature tissue. In addition, LY has a low solubility and tends to clog electrodes. As LY interferes with endogenous electrophysiological properties, it is rather not suitable to be combined with electrophysiological analyses (Tasker et al., 1991). Further fluorophores, e.g., Alexa Fluor (AF) dyes, can be used for tracing coupled cells as well (Han et al., 2013). Like LY, spreading of AF dyes can be assessed directly. However, they do not pass through Cx30 containing gap junctions and just insufficiently through other gap junctions requiring administration at high concentration (Weber et al., 2004). At lower concentrations, there is almost no diffusion to neighboring cells so that AF dyes can be used to label the patch-clamped cell (Augustin et al., 2016;Wadle et al., 2018;Eitelmann et al., 2019). Two other tracers, namely neurobiotin (Nb) and biocytin (Horikawa and Armstrong, 1988;Huang et al., 1992), are colorless and require fixation and further processing of the tissue. Thus, their diffusion cannot be assessed directly. The two tracers are recognized by streptavidin and avidin (Livnah et al., 1993). The latter ones can be linked to either fluorophores or enzymes. Using a fluorophore allows the combination with further immunohistochemical processing of the tissue (Schools et al., 2006;Augustin et al., 2016; Eitelmann et al., 2019, 2020). Using peroxidases produces a light-stable product that is not sensitive to photo-bleaching (Konietzko and Muller, 1994;D’Ambrosio et al., 1998). Alternatively, fluorescent glucose analogues can be used (Speizer et al., 1985;Yamada et al., 2000). They allow to visualize activity-dependent, directed glucose redistribution in otherwise spherical networks (Rouach et al., 2008). However, it has to be kept in mind that, for example, 2-NBDG enters the glycolytic pathway and is degraded to a non-fluorescent derivative (Yoshioka et al., 1996).

**TABLE 1:** Commonly used tracers for analyzing gap junctional coupling.

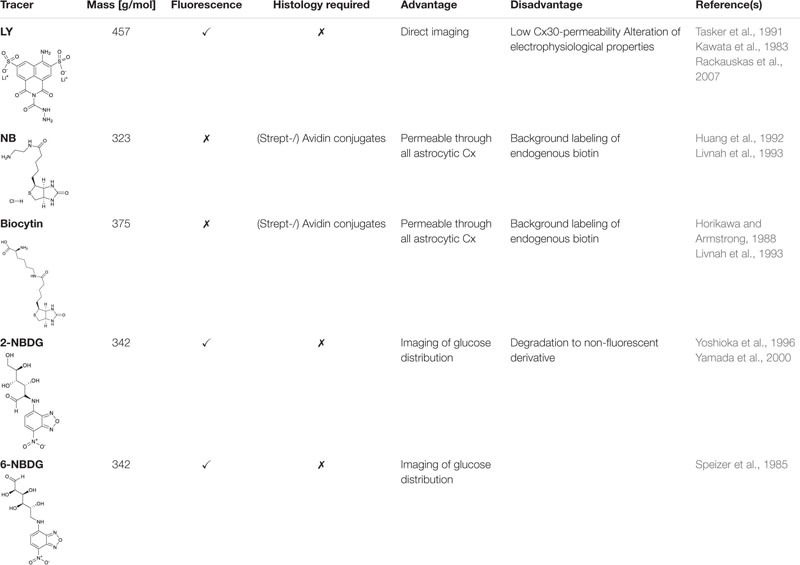

*LY, Lucifer Yellow; NB, neurobiotin; Cx, connexin; NBDG, deoxy-N-(7-nitrobenz-2-oxa-1,3-diazol-4-yl)-aminoglucose; AF, Alexa Fluor.*

**FIGURE 4:**
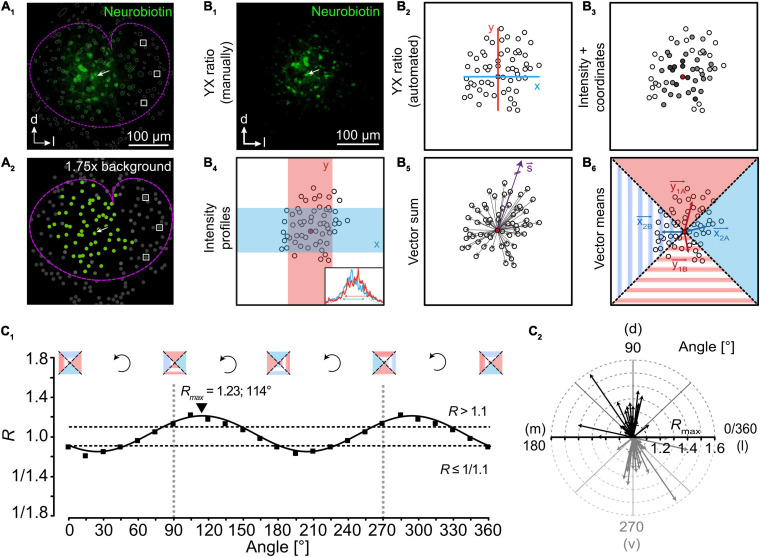
Analysis of network topography. **(A)** Detection of coupled cells. Astrocytes in the lateral superior olive (outlined by dotted magenta line) form large tracer-coupled networks (**A_1_**; green). All identified cells are encircled with a thin gray line. Semi-automated detection of coupled cells is achieved by determining background fluorescence levels of cells (white boxes) being far located from the tracer-filled cell (arrow). Setting a threshold allows for unbiased selection of coupled cells (**A_2_**; green: coupled cells; gray: not coupled cells). **(B)** Approaches to study the topography of tracer-coupled networks. Anisotropy is often determined by the ratio of tracer spread (distance and/or intensity) in two directions that are orthogonal to each other **(B_1–4,6_)**. Alternatively, a purely vector-based approach can be used **(B_5_)**. **(C)** In case of ratio-based analyses, rotation of the coordinate system allows the precise determination of maximal anisotropy and orientation (**C_1_**; exemplarily shown for vector means, **B_6_**). Afterward, results can be gathered in a radar diagram to denote the degree of anisotropy and orientation of networks **(C_2_)**. Figure modified from Eitelmann et al. (2019).

Gap junctional coupling is not uniform, e.g., it was shown to increase developmentally (Binmoller and Muller,1992; Schools et al.,2006; Houades et al.,2008; Langer et al., 2012; Griemsmann et al., 2015). Furthermore, there are many examples of spherical networks upon radial tracer diffusion in certain brain regions (Binmoller and Muller, 1992; Houades et al., 2006; Muller et al., 2009). However, in others—in particular in sensory systems—tracer spreading is unequal in different directions (Houades et al., 2008; Augustin et al., 2016; Claus et al., 2018; Condamine et al., 2018a; Wadle et al., 2018). There, network anisotropy strongly correlates with anatomical and functional organization of the underlying neuronal circuitry. The anisotropy of tracer spreading originates from astrocyte topography (Anders et al., 2014; Augustin et al., 2016; Ghezali et al., 2018; Wadle et al., 2018). Interestingly, in the lateral superior olive—a conspicuous nucleus in the auditory brainstem—absence of spontaneous cochlea-driven neuronal activity leading to disturbed neuronal circuitry (Hirtz et al., 2012; Muller et al., 2019) causes altered astrocyte topography followed by altered orientation of tracer-coupled networks (Eitelmann et al., 2020). In recent years, several different approaches were developed to analyze the anisotropy of tracer-coupled networks (Figure 4). Most approaches are able to reliably detect network anisotropy; however, some are working only in certain brain regions (Eitelmann et al., 2019). The different approaches rely on (1) distance of the farthest cells that are labeled, (2) labeling intensity, (3) position of all coupled cells, or (4) a combination of the aforementioned parameters. Most approaches use the ratio of the diffusion range and/or brightness of the tracer in two directions. The most convenient approach is to measure the extent of tracer spreading in two directions orthogonal to each other (“YX ratio”). Here, anisotropy is determined by the four outermost cells showing tracer signal (Figure 4B_1, 2_; Houades et al., 20062008; Augustin et al., 2016; Ghezali et al., 2018; Wadle et al., 2018). However, since the tracer signal is declining with distance to the loaded cell, it can be difficult to determine the correct extension of the network. Alternatively, the product of network extension multiplied with the somatic tracer intensity for two directions orthogonal to each other can be calculated (“Intensity + coordinates“;Figure 4B_3_;Anders et al., 2014). However, elevated somatic signal intensities due to expression of endogenous biotin (Bixel and Hamprecht, 2000; Yagi et al., 2002) might result in a distorted ratio. In another approach, the labeling intensity of somata and processes is analyzed to determine network anisotropy (“Intensity profiles”;Figure 4B_4_;Claus et al., 2018). Here, intensity plot profiles of two rectangles orthogonal to each other and the ratio of respective full-width at half-maximum (FWHM) are calculated. However, cell number or individual positions are neglected and must be analyzed separately, if required. Finally, there are two vector-based approaches. The first calculates the “Vector sum” (Figure 4B_5_; Condamine et al., 2018a,b). However, it only works in brain regions with defined borders, i.e., diffusion barriers resulting from reduced gap junction coupling. Examples of such brain regions are the trigeminal main sensory nucleus and the columns of the barrel cortex. In both, gap junction coupling is stronger within the nucleus and columns compared to areas outside. However, anisotropic tracer diffusion will be visualized only if the tracer is injected into an astrocyte that is not located in the center (Houades et al., 2008; Condamine et al., 2018a) as the “Vector sum” approach is not capable to detect the anisotropy of tracer-coupled networks if they are symmetric with respect to a point (Eitelmann et al., 2019). The second vector-based approach calculates the “Vector means” in four 90°sectors and the ratio of tracer extension is calculated (Figure 4B_6_; Eitelmann et al., 2019, 2020). However, analyzing the anisotropy of tracer spreading using only two fixed axes might result in falsified results. For example, if an anisotropic network is turned by 45° from one of the two axes, all ratio-based approaches will postulate spherical network. Therefore, a subsequent analysis using a rotating coordinate system will not only determine the maximal anisotropy of a network, but will also define the preferred orientation (Figure 4C;Eitelmann et al., 2019). Taken together, heterogeneity of gap junctional coupling can be visualized excellently by tracer coupling studies. However, they often provide only a snap-shot of coupling using a non-physiological tracer.

### Wide Field (Na^+^) Imaging

**FIGURE 5:**
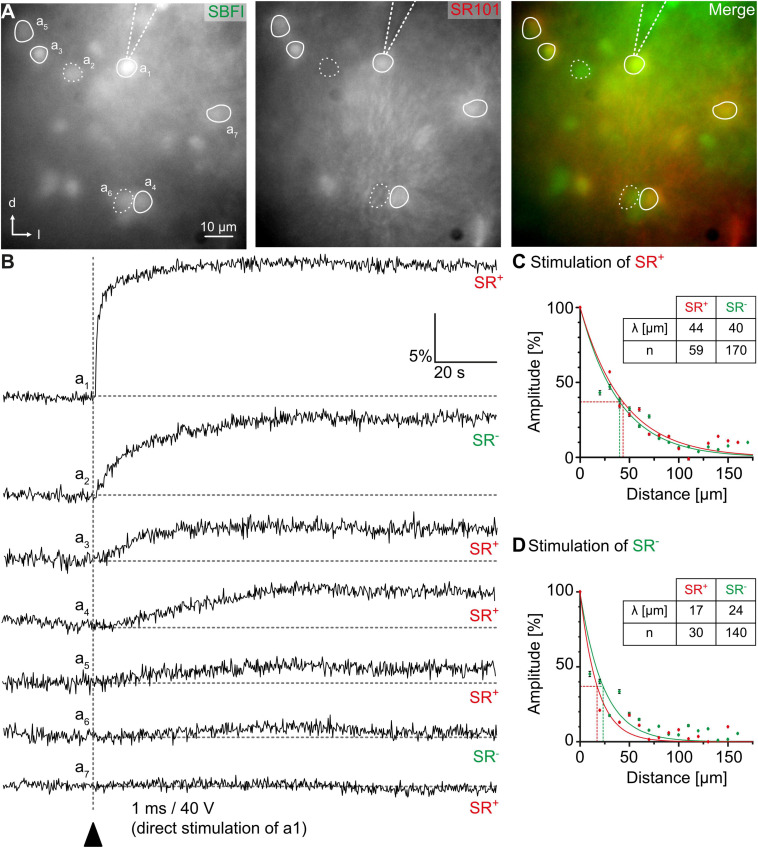
Na^+^ diffusion in glial networks. **(A)** Cells in the center of the inferior colliculus can be loaded with the fluorescent Na^+^ indicator SBFI-AM (left). Astrocytes and *bona fide* oligodendrocytes can be distinguished by sulforhodamine (SR) 101-labeling. **(B)** Electrical stimulation results in Na^+^ load of a single astrocyte **(a_1_)**. Subsequently, neighboring astrocytes (**a_3_–a_5_,a_7_**; SR^+^) and *bona fide* oligodendrocytes (**a_2_,a_6_**; SR^–^), show elevated Na^+^ transients as well. **(C,D)** Na^+^ diffusion can be elicited by stimulation of both astrocytes **(C)** and *bona fide* oligodendrocytes **(D)**. The amplitude of Na^+^ transients depends on the distance from the stimulated cell. Figure modified from Wadle et al. (2018).

Imaging of intracellular ion concentration can be a good tool to supplement tracer coupling studies. For the interpretation of ion diffusion within the gap junction network, it is beneficial to analyze the spread of ions that is less effected by signaling cascades. Thus, Ca^2+^ is a less suitable candidate as signaling to neighboring cells is generated by intra- and extracellular pathways (Giaume and Venance, 1998;Bernardinelli et al., 2004;Scemes and Giaume, 2006). However, intercellular Na^+^ spread depends on gap junctional coupling as deletion of Cx43 and Cx30 prevents ion exchange between neighboring astrocytes (Wallraff et al., 2006;Langer et al., 2012). However, it has to be kept in mind that intracellular Na^+^ is not completely independent from signaling cascades as it is linked to Ca^2+^ via the Na^+^/Ca^2+^ exchanger (Felix et al., 2020a). For Na^+^ imaging, cells are dye-loaded, e.g., with the membrane-permeable form of SBFI (SBFI-acetoxymethyl ester). After cleavage by endogenous esterases SBFI allows ratiometric imaging of [Na^+^]_i_ (Figure 5; Minta and Tsien, 1989;Meier et al., 2006). Na^+^ load into a single cell can be achieved via direct electrical stimulation. This will result in Na^+^ rise in the stimulated and in neighboring cells (Figure 5B; Langer et al., 2012;Augustin et al., 2016;Moshrefi-Ravasdjani et al., 2017;Wadle et al., 2018). Measuring the maximal [Na^+^]_i_ increase allows for the calculation of the length constant λ using a mono-exponential function (Figures 5C,D; Augustin et al., 2016;Moshrefi-Ravasdjani et al., 2017;Wadle et al., 2018), which demonstrates, how efficient Na^+^ is redistributed and how strong gap junctional coupling is. It was shown that spatial spread of Na^+^ between astrocytes was halfway far in the corpus callosum compared to other brain regions, i.e., hippocampus, lateral superior olive, and inferior colliculus (Langer et al., 2012;Augustin et al., 2016;Moshrefi-Ravasdjani et al., 2017;Wadle et al., 2018). Furthermore, Na^+^ diffusion is stronger within homocellular networks. In contrast, in heterocellular (panglial) networks Na^+^ redistribution is reduced (Figure 5D; Moshrefi-Ravasdjani et al., 2017;Wadle et al., 2018), which likely results from a lower permeability of connexons, which are incorporated into the heterotypic gap junction channels (Bedner et al., 2006). Taken together, imaging of ion sensitive dyes is a good supplement to tracer coupling studies to further characterize gap junctional communication.

## Conclusion

Gap junctional communication between glial cells is important for ion and neurotransmitter homeostasis and ensures stabilization of their membrane potential. Though astrocytes throughout the brain share similar properties, they exhibit a considerable amount of region-dependent heterogeneity. To unravel these particular differences suitable approaches are necessary. In this review, we summarized and described well-established and recently developed methods that will allow electrophysiology and imaging laboratories to analyze gap junctional coupling in acute tissue slices.

## Author Contributions

JS and MZ designed the study. JS, MZ, and SE wrote the manuscript. SE designed and arranged the figures. All authors contributed to the article and approved the submitted version.

## Conflict of Interest

The authors declare that the research was conducted in the absence of any commercial or financial relationships that could be construed as a potential conflict of interest.

## References

[B1] Aasen T. Johnstone S. Vidal-Brime L. Lynn K. S. Koval M. (2018). Connexins: synthesis, post-translational modifications, and trafficking in health and disease. *Int J. Mol. Sci.* 19:1296. 10.3390/ijms19051296 PMC5983588 29701678

[B2] Abbaci M. Barberi-Heyob M. Blondel W. Guillemin F. Didelon J. (2008). Advantages and limitations of commonly used methods to assay the molecular permeability of gap junctional intercellular communication. *Biotechniques* 45 56–62. 10.2144/000112810 18611167

[B3] Anders S. Minge D. Griemsmann S. Herde M. K. Steinhauser C. Henneberger C. (2014). Spatial properties of astrocyte gap junction coupling in the rat hippocampus. *Philos. Trans. R. Soc. Lond. B Biol. Sci.* 369:20130600. 10.1098/rstb.2013.0600 PMC4173286 25225094

[B4] Augustin V. Bold C. Wadle S. L. Langer J. Jabs R. Philippot C. (2016). Functional anisotropic panglial networks in the lateral superior olive. *Glia* 64 1892–1911. 10.1002/glia.23031 27458984

[B5] Axelsen L. N. Calloe K. Holstein-Rathlou N. H. Nielsen M. S. (2013). Managing the complexity of communication: regulation of gap junctions by post-translational modification. *Front. Pharmacol.* 4:130. 10.3389/fphar.2013.00130 PMC3804956 24155720

[B6] Bedner P. Dupper A. Huttmann K. Muller J. Herde M. K. Dublin P. (2015). Astrocyte uncoupling as a cause of human temporal lobe epilepsy. *Brain* 138 1208–1222. 10.1093/brain/awv067 25765328 PMC5963418

[B7] Bedner P. Niessen H. Odermatt B. Kretz M. Willecke K. Harz H. (2006). Selective permeability of different connexin channels to the second messenger cyclic AMP. *J. Biol. Chem.* 281 6673–6681. 10.1074/jbc.m511235200 16373337

[B8] Bedner P. Steinhauser C. Theis M. (2012). Functional redundancy and compensation among members of gap junction protein families? *Biochim. Biophys. Acta* 1818 1971–1984. 10.1016/j.bbamem.2011.10.016 22044799

[B9] Bennett M. V. (1966). Physiology of electrotonic junctions. *Ann. N. Y. Acad. Sci.* 137 509–539. 10.1111/j.1749-6632.1966.tb50178.x 5229812

[B10] Bennett M. V. Barrio L. C. Bargiello T. A. Spray D. C. Hertzberg E. Saez J. C. (1991). Gap junctions: new tools, new answers, new questions. *Neuron* 6 305–320. 10.1016/0896-6273(91)90241-q 1848077

[B11] Bernardinelli Y. Magistretti P. J. Chatton J. Y. (2004). Astrocytes generate Na+-mediated metabolic waves. *Proc. Natl. Acad. Sci. U.S.A.* 101 14937–14942. 10.1073/pnas.0405315101 15466714 PMC522032

[B12] Binmoller F. J. Muller C. M. (1992). Postnatal development of dye-coupling among astrocytes in rat visual cortex. *Glia* 6 127–137. 10.1002/glia.440060207 1328051

[B13] Bixel M. G. Hamprecht B. (2000). Immunocytochemical localization of beta-methylcrotonyl-CoA carboxylase in astroglial cells and neurons in culture. *J. Neurochem.* 74 1059–1067. 10.1046/j.1471-4159.2000.0741059.x 10693937

[B14] Breithausen B. Kautzmann S. Boehlen A. Steinhauser C. Henneberger C. (2020). Limited contribution of astroglial gap junction coupling to buffering of extracellular K(+) in CA1 stratum radiatum. *Glia* 68 918–931. 10.1002/glia.23751 31743499

[B15] Bukauskas F. F. Verselis V. K. (2004). Gap junction channel gating. *Biochim. Biophys. Acta* 1662 42–60. 15033578 10.1016/j.bbamem.2004.01.008 PMC2813678

[B16] Cahoy J. D. Emery B. Kaushal A. Foo L. C. Zamanian J. L. Christopherson K. S. (2008). A transcriptome database for astrocytes, neurons, and oligodendrocytes: a new resource for understanding brain development and function. *J. Neurosci.* 28 264–278. 10.1523/jneurosci.4178-07.2008 18171944 PMC6671143

[B17] Chan-Palay V. Palay S. L. (1972). The form of velate astrocytes in the cerebellar cortex of monkey and rat: high voltage electron microscopy of rapid Golgi preparations. *Z. Anat. Entwicklungsgesch* 138 1–19. 10.1007/bf00519921 4629412

[B18] Chaturvedi R. Reddig K. Li H. S. (2014). Long-distance mechanism of neurotransmitter recycling mediated by glial network facilitates visual function in *Drosophila*. *Proc. Natl. Acad. Sci. U.S.A.* 111 2812–2817. 10.1073/pnas.1323714111 24550312 PMC3932938

[B19] Claus L. Philippot C. Griemsmann S. Timmermann A. Jabs R. Henneberger C. (2018). Barreloid borders and neuronal activity shape panglial gap junction-coupled networks in the mouse thalamus. *Cereb. Cortex* 28 213–222. 28095365 10.1093/cercor/bhw368

[B20] Condamine S. Lavoie R. Verdier D. Kolta A. (2018a). Functional rhythmogenic domains defined by astrocytic networks in the trigeminal main sensory nucleus. *Glia* 66 311–326. 10.1002/glia.23244 29058348

[B21] Condamine S. Verdier D. Kolta A. (2018b). Analyzing the size, shape, and directionality of networks of coupled astrocytes. *J. Vis. Exp*. 4 58116. 10.3791/58116 PMC6235419 30346397

[B22] Cotrina M. L. Kang J. Lin J. H. Bueno E. Hansen T. W. He L. (1998). Astrocytic gap junctions remain open during ischemic conditions. *J. Neurosci.* 18 2520–2537. 10.1523/jneurosci.18-07-02520.1998 9502812 PMC6793088

[B23] Cruz N. F. Ball K. K. Dienel G. A. (2007). Functional imaging of focal brain activation in conscious rats: impact of [(14)C]glucose metabolite spreading and release. *J. Neurosci. Res.* 85 3254–3266. 10.1002/jnr.21193 17265468

[B24] Dahl E. Manthey D. Chen Y. Schwarz H. J. Chang Y. S. Lalley P. A. (1996). Molecular cloning and functional expression of mouse connexin-30,a gap junction gene highly expressed in adult brain and skin. *J. Biol. Chem.* 271 17903–17910. 10.1074/jbc.271.30.17903 8663509

[B25] D’Ambrosio R. Wenzel J. Schwartzkroin P. A. Mckhann G. M. II Janigro D. (1998). Functional specialization and topographic segregation of hippocampal astrocytes. *J. Neurosci.* 18 4425–4438. 10.1523/jneurosci.18-12-04425.1998 9614220 PMC4093786

[B26] Droguerre M. Tsurugizawa T. Duchene A. Portal B. Guiard B. P. Deglon N. (2019). A new tool for in vivo study of astrocyte connexin 43 in brain. *Sci. Rep.* 9:18292. 10.1038/s41598-019-54858-9 PMC6892890 31797899

[B27] Du Y. Kiyoshi C. M. Terman D. Zhou M. (2020). “Analysis of the functional states of an astrocyte syncytium,” in *Basic Neurobiology Techniques*, ed. Wright N. (New York, NY: Humana), 285–313. 10.1007/978-1-4939-9944-6_12

[B28] Du Y. Ma B. Kiyoshi C. M. Alford C. C. Wang W. Zhou M. (2015). Freshly dissociated mature hippocampal astrocytes exhibit passive membrane conductance and low membrane resistance similarly to syncytial coupled astrocytes. *J. Neurophysiol.* 113 3744–3750. 10.1152/jn.00206.2015 25810481 PMC4468969

[B29] Egawa K. Yamada J. Furukawa T. Yanagawa Y. Fukuda A. (2013). Cl(-) homeodynamics in gap junction-coupled astrocytic networks on activation of GABAergic synapses. *J. Physiol.* 591 3901–3917. 10.1113/jphysiol.2013.257162 23732644 PMC3764636

[B30] Eitelmann S. Hirtz J. J. Stephan J. (2019). A vector-based method to analyze the topography of glial networks. *Int. J. Mol. Sci.* 20:2821. 10.3390/ijms20112821 PMC6600595 31185593

[B31] Eitelmann S. Petersilie L. Rose C. R. Stephan J. (2020). Altered gap junction network topography in mouse models for human hereditary deafness. *Int. J. Mol. Sci.* 21:7376. 10.3390/ijms21197376 PMC7582522 33036242

[B32] Eng L. F. Ghirnikar R. S. Lee Y. L. (2000). Glial fibrillary acidic protein: GFAP-thirty-one years (1969-2000). *Neurochem. Res.* 25 1439–1451. 11059815 10.1023/a:1007677003387

[B33] Felix L. Delekate A. Petzold G. C. Rose C. R. (2020a). Sodium fluctuations in astroglia and their potential impact on astrocyte function. *Front. Physiol.* 11:871. 10.3389/fphys.2020.00871 PMC7435049 32903427

[B34] Felix L. Stephan J. Rose C. R. (2020b). Astrocytes of the early postnatal brain. *Eur. J. Neurosci*. [Epub ahead of print]. 10.1111/ejn.14780 32406559

[B35] Fuss B. Mallon B. Phan T. Ohlemeyer C. Kirchhoff F. Nishiyama A. (2000). Purification and analysis of in vivo-differentiated oligodendrocytes expressing the green fluorescent protein. *Dev. Biol.* 218 259–274. 10.1006/dbio.1999.9574 10656768

[B36] Ghezali G. Calvo C. F. Pillet L. E. Llense F. Ezan P. Pannasch U. (2018). Connexin 30 controls astroglial polarization during postnatal brain development. *Development* 145:dev155275. 10.1242/dev.155275 PMC5869003 29475972

[B37] Ghirardini E. Wadle S. L. Augustin V. Becker J. Brill S. Hammerich J. (2018). Expression of functional inhibitory neurotransmitter transporters GlyT1, GAT-1, and GAT-3 by astrocytes of inferior colliculus and hippocampus. *Mol. Brain* 11:4. 10.1186/s13041-018-0346-y PMC5785846 29370841

[B38] Giaume C. Koulakoff A. Roux L. Holcman D. Rouach N. (2010). Astroglial networks: a step further in neuroglial and gliovascular interactions. *Nat. Rev. Neurosci.* 11 87–99. 10.1038/nrn2757 20087359

[B39] Giaume C. Saez J. C. Song W. Leybaert L. Naus C. C. (2019). Connexins and pannexins in Alzheimer’s disease. *Neurosci. Lett.* 695 100–105. 10.1016/j.neulet.2017.09.006 28893592

[B40] Giaume C. Theis M. (2010). Pharmacological and genetic approaches to study connexin-mediated channels in glial cells of the central nervous system. *Brain Res. Rev.* 63 160–176. 10.1016/j.brainresrev.2009.11.005 19963007

[B41] Giaume C. Venance L. (1998). Intercellular calcium signaling and gap junctional communication in astrocytes. *Glia* 24 50–64. 10.1002/(sici)1098-1136(199809)24:1<50::aid-glia6>3.0.co;2-4 9700489

[B42] Giaume C. B. Naus C. C. Saez J. C. Leybaert L. (2020). Glial connexins and pannexins in the healthy and diseased brain. *Physiol. Rev*. 101 93–145. 10.1152/physrev.00043.2018 32326824

[B43] Goldberg G. S. Lampe P. D. Nicholson B. J. (1999). Selective transfer of endogenous metabolites through gap junctions composed of different connexins. *Nat. Cell Biol.* 1 457–459. 10.1038/15693 10559992

[B44] Goldberg G. S. Moreno A. P. Lampe P. D. (2002). Gap junctions between cells expressing connexin 43 or 32 show inverse permselectivity to adenosine and ATP. *J. Biol. Chem.* 277 36725–36730. 10.1074/jbc.m109797200 12119284

[B45] Goodenough D. A. Paul D. L. (2009). Gap junctions. *Cold Spring Harb. Perspect. Biol.* 1:a002576. 10.1101/cshperspect.a002576 PMC2742079 20066080

[B46] Griemsmann S. Hoft S. P. Bedner P. Zhang J. Von Staden E. Beinhauer A. (2015). Characterization of panglial gap junction networks in the thalamus, neocortex, and hippocampus reveals a unique population of glial cells. *Cereb. Cortex* 25 3420–3433. 10.1093/cercor/bhu157 25037920 PMC4585496

[B47] Gutierrez Y. Garcia-Marques J. Liu X. Fortes-Marco L. Sanchez-Gonzalez R. Giaume C. (2019). Sibling astrocytes share preferential coupling via gap junctions. *Glia* 67 1852–1858. 10.1002/glia.23662 31216083

[B48] Han X. Chen M. Wang F. Windrem M. Wang S. Shanz S. (2013). Forebrain engraftment by human glial progenitor cells enhances synaptic plasticity and learning in adult mice. *Cell Stem Cell* 12 342–353. 10.1016/j.stem.2012.12.015 23472873 PMC3700554

[B49] Harris A. L. (2007). Connexin channel permeability to cytoplasmic molecules. *Prog. Biophys. Mol. Biol.* 94 120–143. 10.1016/j.pbiomolbio.2007.03.011 17470375 PMC1995164

[B50] Heintz N. (2004). Gene expression nervous system atlas (GENSAT). *Nat. Neurosci.* 7:483. 10.1038/nn0504-483 15114362

[B51] Hille B. (2001). *Ion Channels of Excitable Cells.* Sunderland, MA: Sinauer.

[B52] Hirtz J. J. Braun N. Griesemer D. Hannes C. Janz K. Lohrke S. (2012). Synaptic refinement of an inhibitory topographic map in the auditory brainstem requires functional Cav1.3 calcium channels. *J. Neurosci.* 32 14602–14616. 10.1523/jneurosci.0765-12.2012 23077046 PMC6621425

[B53] Horikawa K. Armstrong W. E. (1988). A versatile means of intracellular labeling: injection of biocytin and its detection with avidin conjugates. *J. Neurosci. Methods* 25 1–11. 10.1016/0165-0270(88)90114-8 3146670

[B54] Houades V. Koulakoff A. Ezan P. Seif I. Giaume C. (2008). Gap junction-mediated astrocytic networks in the mouse barrel cortex. *J. Neurosci.* 28 5207–5217. 10.1523/jneurosci.5100-07.2008 18480277 PMC6670639

[B55] Houades V. Rouach N. Ezan P. Kirchhoff F. Koulakoff A. Giaume C. (2006). Shapes of astrocyte networks in the juvenile brain. *Neuron Glia Biol.* 2 3–14. 10.1017/s1740925x06000081 18634587

[B56] Huang M. Du Y. Kiyoshi C. Wu X. Askwith C. Mctigue D. (2018). Syncytial isopotentiality: an electrical feature of spinal cord astrocyte networks. *Neuroglia* 1 271–279. 10.3390/neuroglia1010018

[B57] Huang Q. Zhou D. Difiglia M. (1992). Neurobiotin, a useful neuroanatomical tracer for in vivo anterograde, retrograde and transneuronal tract-tracing and for in vitro labeling of neurons. *J. Neurosci. Methods* 41 31–43. 10.1016/0165-0270(92)90121-s 1578900

[B58] Kafitz K. W. Meier S. D. Stephan J. Rose C. R. (2008). Developmental profile and properties of sulforhodamine 101–Labeled glial cells in acute brain slices of rat hippocampus. *J. Neurosci. Methods* 169 84–92. 10.1016/j.jneumeth.2007.11.022 18187203

[B59] Kang B. E. Lee S. Baker B. J. (2019). Optical consequences of a genetically-encoded voltage indicator with a pH sensitive fluorescent protein. *Neurosci. Res.* 146 13–21. 10.1016/j.neures.2018.10.006 30342069 PMC6470055

[B60] Karram K. Goebbels S. Schwab M. Jennissen K. Seifert G. Steinhauser C. (2008). NG2-expressing cells in the nervous system revealed by the NG2-EYFP-knockin mouse. *Genesis* 46 743–757. 10.1002/dvg.20440 18924152

[B61] Kawata M. Sano Y. Inenaga K. Yamashita H. (1983). Immunohistochemical identification of lucifer yellow-labeled neurons in the rat supraoptic nucleus. *Histochemistry* 78 21–26. 10.1007/bf00491107 6347986

[B62] Kettenmann H. Ransom B. R. (1988). Electrical coupling between astrocytes and between oligodendrocytes studied in mammalian cell cultures. *Glia* 1 64–73. 10.1002/glia.440010108 2853139

[B63] Kita Y. Kawakami K. Takahashi Y. Murakami F. (2013). Development of cerebellar neurons and glias revealed by in utero electroporation: golgi-like labeling of cerebellar neurons and glias. *PLoS One* 8:e70091. 10.1371/journal.pone.0070091 PMC3720936 23894597

[B64] Kiyoshi C. M. Aten S. Arzola E. P. Patterson J. A. Taylor A. T. Du Y. (2020). Ultrastructural view of astrocyte-astrocyte and astrocyte-synapse contacts within the hippocampus. *BioRxiv* 10.1101/2020.10.28.358200 PMC9050955 35283239

[B65] Kiyoshi C. M. Du Y. Zhong S. Wang W. Taylor A. T. Xiong B. (2018). Syncytial isopotentiality: a system-wide electrical feature of astrocytic networks in the brain. *Glia* 66 2756–2769. 10.1002/glia.23525 30277621 PMC8818325

[B66] Konietzko U. Muller C. M. (1994). Astrocytic dye coupling in rat hippocampus: topography, developmental onset, and modulation by protein kinase C. *Hippocampus* 4 297–306. 10.1002/hipo.450040313 7842053

[B67] Kuffler S. W. Nicholls J. G. Orkand R. K. (1966). Physiological properties of glial cells in the central nervous system of amphibia. *J. Neurophysiol.* 29 768–787. 10.1152/jn.1966.29.4.768 5966434

[B68] Kunzelmann P. Schroder W. Traub O. Steinhauser C. Dermietzel R. Willecke K. (1999). Late onset and increasing expression of the gap junction protein connexin30 in adult murine brain and long-term cultured astrocytes. *Glia* 25 111–119. 10.1002/(sici)1098-1136(19990115)25:2<111::aid-glia2>3.0.co;2-i 9890626

[B69] Langer J. Stephan J. Theis M. Rose C. R. (2012). Gap junctions mediate intercellular spread of sodium between hippocampal astrocytes in situ. *Glia* 60 239–252. 10.1002/glia.21259 22025386

[B70] Lee C. Y. Dallerac G. Ezan P. Anderova M. Rouach N. (2016). Glucose tightly controls morphological and functional properties of astrocytes. *Front. Aging Neurosci.* 8:82. 10.3389/fnagi.2016.00082 PMC4834307 27148048

[B71] Livnah O. Bayer E. A. Wilchek M. Sussman J. L. (1993). Three-dimensional structures of avidin and the avidin-biotin complex. *Proc. Natl. Acad. Sci. U.S.A.* 90 5076–5080. 10.1073/pnas.90.11.5076 8506353 PMC46657

[B72] Lunde L. K. Camassa L. M. Hoddevik E. H. Khan F. H. Ottersen O. P. Boldt H. B. (2015). Postnatal development of the molecular complex underlying astrocyte polarization. *Brain Struct. Funct.* 220 2087–2101. 10.1007/s00429-014-0775-z 24777283 PMC4481305

[B73] Ma B. Buckalew R. Du Y. Kiyoshi C. M. Alford C. C. Wang W. (2016). Gap junction coupling confers isopotentiality on astrocyte syncytium. *Glia* 64 214–226. 10.1002/glia.22924 26435164 PMC4595908

[B74] MacAulay N. (2020). Molecular mechanisms of K(+) clearance and extracellular space shrinkage-Glia cells as the stars. *Glia* 68 2192–2211. 32181522 10.1002/glia.23824

[B75] Maglione M. Tress O. Haas B. Karram K. Trotter J. Willecke K. (2010). Oligodendrocytes in mouse corpus callosum are coupled via gap junction channels formed by connexin47 and connexin32. *Glia* 58 1104–1117. 10.1002/glia.20991 20468052

[B76] Matthias K. Kirchhoff F. Seifert G. Huttmann K. Matyash M. Kettenmann H. (2003). Segregated expression of AMPA-type glutamate receptors and glutamate transporters defines distinct astrocyte populations in the mouse hippocampus. *J. Neurosci.* 23 1750–1758. 10.1523/jneurosci.23-05-01750.2003 12629179 PMC6741945

[B77] McCutcheon S. Stout R. F. Jr. Spray D. C. (2020). The dynamic Nexus: gap junctions control protein localization and mobility in distinct and surprising ways. *Sci. Rep.* 10:17011. 10.1038/s41598-020-73892-6 PMC7550573 33046777

[B78] Meier S. D. Kovalchuk Y. Rose C. R. (2006). Properties of the new fluorescent Na+ indicator CoroNa Green: comparison with SBFI and confocal Na+ imaging. *J. Neurosci. Methods* 155 251–259. 10.1016/j.jneumeth.2006.01.009 16488020

[B79] Meme W. Vandecasteele M. Giaume C. Venance L. (2009). Electrical coupling between hippocampal astrocytes in rat brain slices. *Neurosci. Res.* 63 236–243. 10.1016/j.neures.2008.12.008 19167439

[B80] Minta A. Tsien R. Y. (1989). Fluorescent indicators for cytosolic sodium. *J. Biol. Chem.* 264 19449–19457. 10.1016/s0021-9258(19)47321-3 2808435

[B81] Moroni R. F. Inverardi F. Regondi M. C. Pennacchio P. Frassoni C. (2015). Developmental expression of Kir4.1 in astrocytes and oligodendrocytes of rat somatosensory cortex and hippocampus. *Int. J. Dev. Neurosci.* 47 198–205. 10.1016/j.ijdevneu.2015.09.004 26427731

[B82] Moshrefi-Ravasdjani B. Hammel E. L. Kafitz K. W. Rose C. R. (2017). Astrocyte sodium signalling and panglial spread of sodium signals in brain white matter. *Neurochem. Res.* 42 2505–2518. 10.1007/s11064-017-2197-9 28214986

[B83] Muller C. M. (1996). “Gap-junctional communication in mammalian cortical astrocytes: development, modifiability and possible functions,” in *Gap Junctions in the Nervous System*, eds Spary D. C. Dermietzel R. (Austin, TX: RG Landes Company), 203–212. 10.1007/978-3-662-21935-5_12

[B84] Muller J. Reyes-Haro D. Pivneva T. Nolte C. Schaette R. Lubke J. (2009). The principal neurons of the medial nucleus of the trapezoid body and NG2(+) glial cells receive coordinated excitatory synaptic input. *J. Gen. Physiol.* 134 115–127. 10.1085/jgp.200910194 19635853 PMC2717692

[B85] Muller N. I. C. Sonntag M. Maraslioglu A. Hirtz J. J. Friauf E. (2019). Topographic map refinement and synaptic strengthening of a sound localization circuit require spontaneous peripheral activity. *J. Physiol.* 597 5469–5493. 10.1113/jp277757 31529505

[B86] Muller T. Moller T. Neuhaus J. Kettenmann H. (1996). Electrical coupling among Bergmann glial cells and its modulation by glutamate receptor activation. *Glia* 17 274–284. 10.1002/(sici)1098-1136(199608)17:4<274::aid-glia2>3.0.co;2-# 8856324

[B87] Murphy A. D. Hadley R. D. Kater S. B. (1983). Axotomy-induced parallel increases in electrical and dye coupling between identified neurons of Helisoma. *J. Neurosci.* 3 1422–1429. 10.1523/jneurosci.03-07-01422.1983 6306177 PMC6564434

[B88] Nagy J. I. Patel D. Ochalski P. A. Stelmack G. L. (1999). Connexin30 in rodent, cat and human brain: selective expression in gray matter astrocytes, co-localization with connexin43 at gap junctions and late developmental appearance. *Neuroscience* 88 447–468. 10.1016/s0306-4522(98)00191-2 10197766

[B89] Nagy J. I. Rash J. E. (2000). Connexins and gap junctions of astrocytes and oligodendrocytes in the CNS. *Brain Res. Brain Res. Rev.* 32 29–44. 10.1016/s0165-0173(99)00066-1 10751655

[B90] Nakase T. Yoshida Y. Nagata K. (2006). Enhanced connexin 43 immunoreactivity in penumbral areas in the human brain following ischemia. *Glia* 54 369–375. 10.1002/glia.20399 16886200

[B91] Nielsen M. S. Axelsen L. N. Sorgen P. L. Verma V. Delmar M. Holstein-Rathlou N. H. (2012). Gap junctions. *Compr. Physiol.* 2 1981–2035. 23723031 10.1002/cphy.c110051 PMC3821273

[B92] Niessen H. Harz H. Bedner P. Kramer K. Willecke K. (2000). Selective permeability of different connexin channels to the second messenger inositol 1,4,5-trisphosphate. *J. Cell Sci.* 113(Pt 8), 1365–1372. 10725220 10.1242/jcs.113.8.1365

[B93] Nimmerjahn A. Kirchhoff F. Kerr J. N. Helmchen F. (2004). Sulforhodamine 101 as a specific marker of astroglia in the neocortex in vivo. *Nat. Methods* 1 31–37. 10.1038/nmeth706 15782150

[B94] Nolte C. Matyash M. Pivneva T. Schipke C. G. Ohlemeyer C. Hanisch U. K. (2001). GFAP promoter-controlled EGFP-expressing transgenic mice: a tool to visualize astrocytes and astrogliosis in living brain tissue. *Glia* 33 72–86. 10.1002/1098-1136(20010101)33:1<72::aid-glia1007>3.0.co;2-a 11169793

[B95] Nwaobi S. E. Lin E. Peramsetty S. R. Olsen M. L. (2014). DNA methylation functions as a critical regulator of Kir4.1 expression during CNS development. *Glia* 62 411–427. 10.1002/glia.22613 24415225 PMC3991476

[B96] Olsen M. L. Khakh B. S. Skatchkov S. N. Zhou M. Lee C. J. Rouach N. (2015). New insights on astrocyte ion channels: critical for homeostasis and neuron-glia signaling. *J. Neurosci.* 35 13827–13835. 10.1523/jneurosci.2603-15.2015 26468182 PMC4604221

[B97] Pannasch U. Vargova L. Reingruber J. Ezan P. Holcman D. Giaume C. (2011). Astroglial networks scale synaptic activity and plasticity. *Proc. Natl. Acad. Sci. U.S.A.* 108 8467–8472. 10.1073/pnas.1016650108 21536893 PMC3100942

[B98] Rackauskas M. Verselis V. K. Bukauskas F. F. (2007). Permeability of homotypic and heterotypic gap junction channels formed of cardiac connexins mCx30.2, *Cx*40, Cx43, and Cx45. *Am. J. Physiol. Heart Circ. Physiol.* 293 H1729–H1736. 17557922 10.1152/ajpheart.00234.2007 PMC2836796

[B99] Ransom B. R. Goldring S. (1973). Ionic determinants of membrane potential of cells presumed to be glia in cerebral cortex of cat. *J. Neurophysiol.* 36 855–868. 10.1152/jn.1973.36.5.855 4805015

[B100] Ransom B. R. Kettenmann H. (1990). Electrical coupling, without dye coupling, between mammalian astrocytes and oligodendrocytes in cell culture. *Glia* 3 258–266. 10.1002/glia.440030405 2144505

[B101] Rouach N. Koulakoff A. Abudara V. Willecke K. Giaume C. (2008). Astroglial metabolic networks sustain hippocampal synaptic transmission. *Science* 322 1551–1555. 10.1126/science.1164022 19056987

[B102] Scemes E. Giaume C. (2006). Astrocyte calcium waves: what they are and what they do. *Glia* 54 716–725. 10.1002/glia.20374 17006900 PMC2605018

[B103] Schools G. P. Zhou M. Kimelberg H. K. (2006). Development of gap junctions in hippocampal astrocytes: evidence that whole cell electrophysiological phenotype is an intrinsic property of the individual cell. *J. Neurophysiol.* 96 1383–1392. 10.1152/jn.00449.2006 16775204

[B104] Schreiner A. E. Durry S. Aida T. Stock M. C. Ruther U. Tanaka K. (2014). Laminar and subcellular heterogeneity of GLAST and GLT-1 immunoreactivity in the developing postnatal mouse hippocampus. *J. Comp. Neurol.* 522 204–224. 10.1002/cne.23450 23939750

[B105] Seifert G. Huttmann K. Binder D. K. Hartmann C. Wyczynski A. Neusch C. (2009). Analysis of astroglial K+ channel expression in the developing hippocampus reveals a predominant role of the Kir4.1 subunit. *J. Neurosci.* 29 7474–7488. 10.1523/jneurosci.3790-08.2009 19515915 PMC6665420

[B106] Sontheimer H. Waxman S. G. Ransom B. R. (1991). Relationship between Na+ current expression and cell-cell coupling in astrocytes cultured from rat hippocampus. *J. Neurophysiol.* 65 989–1002. 10.1152/jn.1991.65.4.989 2051214

[B107] Speizer L. Haugland R. Kutchai H. (1985). Asymmetric transport of a fluorescent glucose analogue by human erythrocytes. *Biochim. Biophys. Acta* 815 75–84. 10.1016/0005-2736(85)90476-6 4039191

[B108] Stephan J. Friauf E. (2014). Functional analysis of the inhibitory neurotransmitter transporters GlyT1, GAT-1, and GAT-3 in astrocytes of the lateral superior olive. *Glia* 62 1992–2003. 10.1002/glia.22720 25103283

[B109] Stephan J. Haack N. Kafitz K. W. Durry S. Koch D. Hochstrate P. (2012). Kir4.1 channels mediate a depolarization of hippocampal astrocytes under hyperammonemic conditions in situ. *Glia* 60 965–978. 10.1002/glia.22328 22431254

[B110] Susaki E. A. Tainaka K. Perrin D. Kishino F. Tawara T. Watanabe T. M. (2014). Whole-brain imaging with single-cell resolution using chemical cocktails and computational analysis. *Cell* 157 726–739. 10.1016/j.cell.2014.03.042 24746791

[B111] Susaki E. A. Tainaka K. Perrin D. Yukinaga H. Kuno A. Ueda H. R. (2015). Advanced CUBIC protocols for whole-brain and whole-body clearing and imaging. *Nat. Protoc.* 10 1709–1727. 10.1038/nprot.2015.085 26448360

[B112] Tasker J. G. Hoffman N. W. Dudek F. E. (1991). Comparison of three intracellular markers for combined electrophysiological, morphological and immunohistochemical analyses. *J. Neurosci. Methods* 38 129–143. 10.1016/0165-0270(91)90163-t 1723776

[B113] Terman D. Zhou M. (2019). Modeling the role of the astrocyte syncytium and K+ buffering in maintaining neuronal firing patterns. *Opera Med. Physiol.* 5 7–16.

[B114] Veenstra R. D. (1996). Size and selectivity of gap junction channels formed from different connexins. *J. Bioenerg. Biomembr.* 28 327–337. 10.1007/bf02110109 8844330

[B115] Veenstra R. D. Wang H. Z. Beblo D. A. Chilton M. G. Harris A. L. Beyer E. C. (1995). Selectivity of connexin-specific gap junctions does not correlate with channel conductance. *Circ. Res.* 77 1156–1165. 10.1161/01.res.77.6.1156 7586229

[B116] Wadle S. L. Augustin V. Langer J. Jabs R. Philippot C. Weingarten D. J. (2018). Anisotropic panglial coupling reflects tonotopic organization in the inferior colliculus. *Front. Cell Neurosci.* 12:431. 10.3389/fncel.2018.00431 PMC6277822 30542265

[B117] Wallraff A. Kohling R. Heinemann U. Theis M. Willecke K. Steinhauser C. (2006). The impact of astrocytic gap junctional coupling on potassium buffering in the hippocampus. *J. Neurosci.* 26 5438–5447. 10.1523/jneurosci.0037-06.2006 16707796 PMC6675300

[B118] Wallraff A. Odermatt B. Willecke K. Steinhauser C. (2004). Distinct types of astroglial cells in the hippocampus differ in gap junction coupling. *Glia* 48 36–43. 10.1002/glia.20040 15326613

[B119] Wang H. Z. Veenstra R. D. (1997). Monovalent ion selectivity sequences of the rat connexin43 gap junction channel. *J. Gen. Physiol.* 109 491–507. 10.1085/jgp.109.4.491 9101407 PMC2219435

[B120] Wang Q. Wang W. Aten S. Kiyoshi C. M. Du Y. Zhou M. (2020). Epileptiform neuronal discharges impair astrocyte syncytial isopotentiality in acute hippocampal slices. *Brain Sci.* 10:208. 10.3390/brainsci10040208 PMC7226063 32252295

[B121] Wang W. Kiyoshi C. M. Du Y. Taylor A. T. Sheehan E. R. Wu X. (2020). TREK-1 null impairs neuronal excitability, synaptic plasticity, and cognitive function. *Mol. Neurobiol.* 57 1332–1346. 10.1007/s12035-019-01828-x 31728930 PMC8808335

[B122] Wasseff S. K. Scherer S. S. (2011). Cx32 and Cx47 mediate oligodendrocyte:astrocyte and oligodendrocyte:oligodendrocyte gap junction coupling. *Neurobiol. Dis.* 42 506–513. 10.1016/j.nbd.2011.03.003 21396451 PMC3773476

[B123] Weber P. A. Chang H. C. Spaeth K. E. Nitsche J. M. Nicholson B. J. (2004). The permeability of gap junction channels to probes of different size is dependent on connexin composition and permeant-pore affinities. *Biophys. J.* 87 958–973. 10.1529/biophysj.103.036350 15298902 PMC1304503

[B124] Wu L. Dong A. Dong L. Wang S. Q. Li Y. (2019). PARIS, an optogenetic method for functionally mapping gap junctions. *eLife* 8:e43366. 10.7554/eLife.43366 PMC6396999 30638447

[B125] Xin W. Schuebel K. E. Jair K. W. Cimbro R. De Biase L. M. Goldman D. (2019). Ventral midbrain astrocytes display unique physiological features and sensitivity to dopamine D2 receptor signaling. *Neuropsychopharmacology* 44 344–355. 10.1038/s41386-018-0151-4 30054584 PMC6300565

[B126] Xu G. Wang W. Kimelberg H. K. Zhou M. (2010). Electrical coupling of astrocytes in rat hippocampal slices under physiological and simulated ischemic conditions. *Glia* 58 481–493. 19795502 10.1002/glia.20939

[B127] Xu G. Wang W. Zhou M. (2014). Spatial organization of NG2 glial cells and astrocytes in rat hippocampal CA1 region. *Hippocampus* 24 383–395. 10.1002/hipo.22232 24339242 PMC3971479

[B128] Yagi T. Terada N. Baba T. Ohno S. (2002). Localization of endogenous biotin-containing proteins in mouse Bergmann glial cells. *Histochem. J.* 34 567–572. 14626347 10.1023/a:1026053029546

[B129] Yamada K. Nakata M. Horimoto N. Saito M. Matsuoka H. Inagaki N. (2000). Measurement of glucose uptake and intracellular calcium concentration in single, living pancreatic beta-cells. *J. Biol. Chem.* 275 22278–22283. 10.1074/jbc.m908048199 10748091

[B130] Yang Y. Vidensky S. Jin L. Jie C. Lorenzini I. Frankl M. (2011). Molecular comparison of GLT1+ and ALDH1L1+ astrocytes in vivo in astroglial reporter mice. *Glia* 59 200–207. 10.1002/glia.21089 21046559 PMC3199134

[B131] Yoshioka K. Saito M. Oh K. B. Nemoto Y. Matsuoka H. Natsume M. (1996). Intracellular fate of 2-NBDG, a fluorescent probe for glucose uptake activity, in *Escherichia coli* cells. *Biosci. Biotechnol. Biochem.* 60 1899–1901. 10.1271/bbb.60.1899 8987871

[B132] Zhong S. Du Y. Kiyoshi C. M. Ma B. Alford C. C. Wang Q. (2016). Electrophysiological behavior of neonatal astrocytes in hippocampal stratum radiatum. *Mol. Brain* 9:34. 10.1186/s13041-016-0213-7 PMC4802662 27004553

[B133] Zhou M. Schools G. P. Kimelberg H. K. (2006). Development of GLAST(+) astrocytes and NG2(+) glia in rat hippocampus CA1: mature astrocytes are electrophysiologically passive. *J. Neurophysiol.* 95 134–143. 10.1152/jn.00570.2005 16093329

